# First person – Sarah Elzinga

**DOI:** 10.1242/dmm.049033

**Published:** 2021-04-15

## Abstract

First Person is a series of interviews with the first authors of a selection of papers published in Disease Models & Mechanisms, helping early-career researchers promote themselves alongside their papers. Sarah Elzinga is first author on ‘Sex differences in insulin resistance, but not peripheral neuropathy, in a diet-induced prediabetes mouse model’, published in DMM. Sarah is a postdoc in the lab of Eva Feldman at the University of Michigan, Ann Arbor, MI, USA, investigating immune-mediated mechanisms that promote neurodegeneration in obesity, metabolic syndrome, prediabetes and type 2 diabetes.


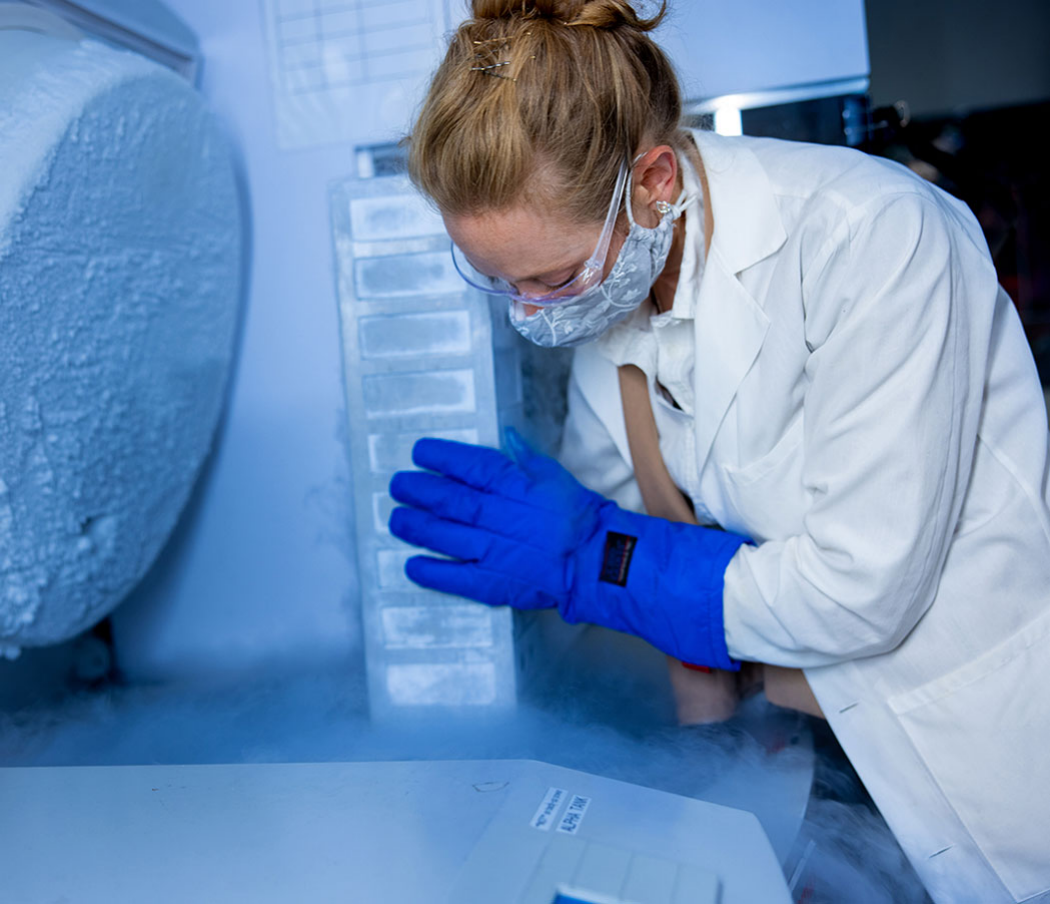


**Sarah Elzinga**

**How would you explain the main findings of your paper to non-scientific family and friends?**

We found that female mice fed a high-fat diet (HFD) gained weight more slowly and had a delay in their development of insulin resistance compared to males fed the same HFD. However, both male and female mice fed a HFD developed peripheral neuropathy to a similar extent and on a similar timeline, as measured by slower nerve conduction velocities and fewer nerves innervating the skin on their foot pads.

**What are the potential implications of these results for your field of research?**

These results indicate that HFD in mice is a good model for human diabetic peripheral neuropathy for both males and females, and is useful to more fully understand the complex relationship between metabolism and sex in peripheral neuropathy.

**What are the main advantages and drawbacks of the model system you have used as it relates to the disease you are investigating?**

While we believe HFD-fed mice are a good model for peripheral neuropathy, as these animals develop reproducible and robust obesity, prediabetes and neuropathy, they do present some drawbacks. First, mice never perfectly model human disease. Second, while HFD-fed mice develop prediabetes, they do not progress to frank type 2 diabetes as is often the case in humans. Finally, lipid profiles in HFD mice do not faithfully mimic those in man, making lipid-related changes more difficult to interpret translationally.

**What has surprised you the most while conducting your research?**

Given the protective metabolic effects observed in females, we expected to see a similar protective effect on their peripheral neuropathy.

**Describe what you think is the most significant challenge impacting your research at this time and how will this be addressed over the next 10 years?**

The most significant challenge to my research is to identify the underlying inflammatory mechanisms that contribute to neurodegeneration so that we can create much needed targeted treatments and treatment strategies for these conditions.

**Figure:**
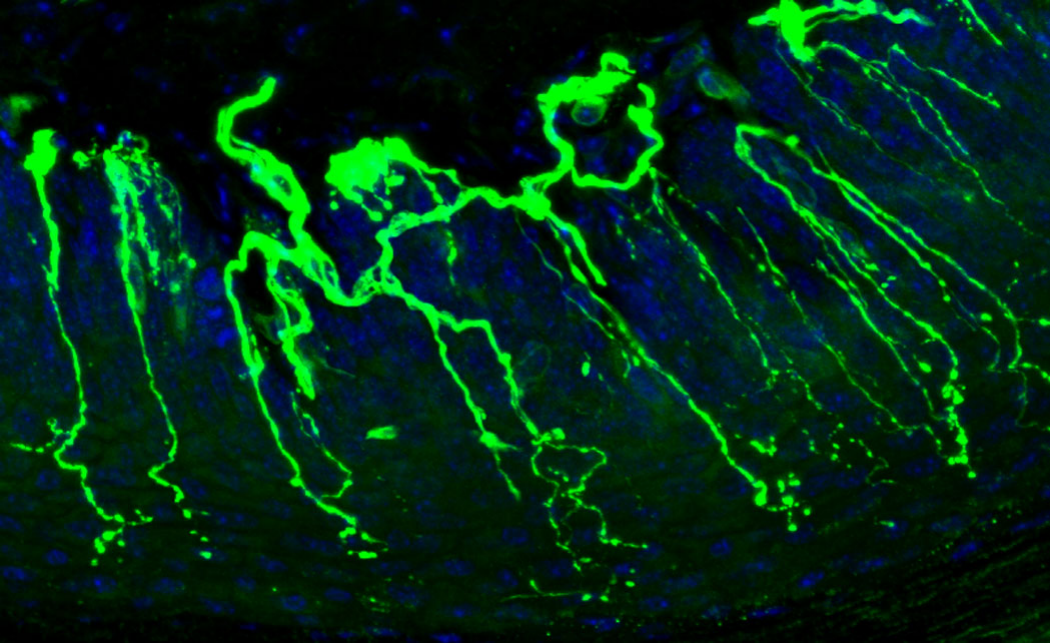
**Nerve fibres (green) penetrating the epidermis of a mouse foot pad used to help determine small-fibre neuropathy.**

“[…] supporting scientists early in their careers is vital to the future of research.”

**What changes do you think could improve the professional lives of early-career scientists?**

I believe that supporting scientists early in their careers is vital to the future of research. Obtaining funding and publications without the benefit of years of research is often a very difficult task, and many talented new scientists can end up leaving their fields because of this.

**What's next for you?**

The next steps for me are to complete my postdoctoral training and start an independent laboratory of my own.
